# Altered Intracellular Calcium Homeostasis and Arrhythmogenesis in the Aged Heart

**DOI:** 10.3390/ijms20102386

**Published:** 2019-05-14

**Authors:** Shanna Hamilton, Dmitry Terentyev

**Affiliations:** 1 Dorothy M. Davis Heart and Lung Research Institute, College of Medicine, The Ohio State University, Columbus, OH 43210, USA; shanna.hamilton@osumc.edu; 2 Department of Physiology and Cell Biology, College of Medicine, The Ohio State University, Columbus, OH 43210, USA

**Keywords:** calcium signaling, cardiac arrhythmia, ageing, ryanodine receptor, sarco/endoplasmic Ca^2+^-ATPase

## Abstract

Aging of the heart is associated with a blunted response to sympathetic stimulation, reduced contractility, and increased propensity for arrhythmias, with the risk of sudden cardiac death significantly increased in the elderly population. The altered cardiac structural and functional phenotype, as well as age-associated prevalent comorbidities including hypertension and atherosclerosis, predispose the heart to atrial fibrillation, heart failure, and ventricular tachyarrhythmias. At the cellular level, perturbations in mitochondrial function, excitation-contraction coupling, and calcium homeostasis contribute to this electrical and contractile dysfunction. Major determinants of cardiac contractility are the intracellular release of Ca^2+^ from the sarcoplasmic reticulum by the ryanodine receptors (RyR2), and the following sequestration of Ca^2+^ by the sarco/endoplasmic Ca^2+^-ATPase (SERCa2a). Activity of RyR2 and SERCa2a in myocytes is not only dependent on expression levels and interacting accessory proteins, but on fine-tuned regulation via post-translational modifications. In this paper, we review how aberrant changes in intracellular Ca^2+^ cycling via these proteins contributes to arrhythmogenesis in the aged heart.

## 1. Introduction

Heart disease remains the leading cause of death in the US [1], in part due to the aging population. There is a significantly increased incidence of cardiovascular disease and arrhythmia in the elderly [2,3], and over 130 million adults are projected to have a form of cardiac disease by 2035, with costs to the economy expected to top $1 trillion [1]. An understanding of the molecular and cellular mechanisms underlying arrhythmogenesis in aging remains paramount for the development of targeted therapeutics that may reduce this burden.

At the cellular level, the cardiac disease phenotype is a culmination of altered response to β-adrenergic stimulation [4,5,6], mitochondrial dysfunction [7], increased reactive oxygen species (ROS) emission [8,9], and dysregulated Ca^2+^ homeostasis [10]. This causes impaired systolic and diastolic function, impaired relaxation, and cardiac arrhythmia. 

Contraction and relaxation of the cardiomyocyte is driven by precise, coordinated excitation-contraction coupling, which is the process linking electrical excitability and intracellular Ca^2+^ homeostasis. Triggering action potentials depolarize the cell and open L-type Ca^2+^ channels (LTCCs) at the sarcolemma, which initiates Ca^2+^ release from the sarcoplasmic reticulum (SR) Ca^2+^ release channels and the cardiac ryanodine receptors (RyR2s). Global increases in intracellular Ca^2+^ concentration ([Ca^2+^]_i_) activate contractile machinery, which initiates cardiac systole. Relaxation and diastole ensue when Ca^2+^ is extruded from the cytosol via the Na^+^/Ca^2+^ exchanger (NCX1, major cardiac isoform) and sequestered into the SR via the sarco/endoplasmic reticulum Ca^2+^-ATPase (SERCa2a, major cardiac isoform). Any perturbations in intracellular Ca^2+^ handling can, therefore, alter contractility and electrical stability of the heart. It is well established that excitation-contraction coupling becomes dysfunctional with age, which drives increased propensity for cardiac arrhythmias in the elderly population [10,11]. 

We and others have comprehensively reviewed the function, post-translational modifications, and role of excitation-contraction coupling proteins in the development of cardiac arrhythmia [12,13,14,15,16]. In this case, we provide a more concentrated focus on aberrant intracellular Ca^2+^ release and mechanisms of arrhythmogenesis that occur in the aged heart. 

## 2. Intracellular Ca^2+^ Homeostasis in the Aged Heart

### 2.1. Ryanodine Receptor

Much of the intracellular Ca^2+^ required for cardiac contraction comes via the major intracellular SR Ca^2+^ release channel, RyR2 [17]. Within junctional SR, RyR2 channels are in close proximity of LTCCs to facilitate rapid and coordinated Ca^2+^ transport upon sarcolemma depolarization. A small LTCC-mediated influx of Ca^2+^ into the cytosol activates single clusters of RyR2 channels. The subsequent increase in local [Ca^2+^]_i_, known as a Ca^2+^ spark [18], activates other clusters of RyR2 channels and triggers a much larger Ca^2+^-induced Ca^2+^ release (CICR) from the SR across the cardiomyocyte [19]. The summation of Ca^2+^ sparks results in a global increase in [Ca^2+^]_i_, or Ca^2+^ transient, which initiates muscle contraction.

The open probability of RyR2 channels is finite, which leads to small and unsynchronized SR Ca^2+^ release events during diastole known as SR Ca^2+^ leak [20]. Some of this leak can be visualized as Ca^2+^ sparks, while some is effectively invisible due to current detection limits. Although leak plays an important physiological role in determining SR Ca^2+^ content, an enhanced leak can be detrimental to cardiac function [13,16,21]. Increased spontaneous Ca^2+^ leak during diastole promotes an untimely inward depolarizing current via NCX1, which leads to early and delayed afterdepolarizations (EADs and DADs, respectively) and the initiation of triggered activity. A reduction of SR Ca^2+^ release during systole also contributes to diminished contractile function, which predisposes the heart to arrhythmogenesis [14,21,22]. 

Leak is elevated in the failing heart [23], as well as in conditions characterized by gain-of-function mutations in proteins of the RyR2 macromolecular complex, such as catecholaminergic polymorphic ventricular tachycardia (CPVT) [24,25]. It is also evident that Ca^2+^ leak is elevated in the aging heart [9,26,27,28]. The propensity for diastolic Ca^2+^ sparks and waves was shown to be significantly increased in aged mice, while stabilization of interdomain interactions in RyR2 via application of therapeutic dantrolene could reduce excessive SR Ca^2+^ leak and attenuate these pro-arrhythmic events [28]. In aged rat ventricular myocytes, Zhu et al. 2005 reported a decreased Ca^2+^ transient amplitude and a reduced SR Ca^2+^ content, as well as an increased spontaneous Ca^2+^ spark frequency with a reduced average amplitude [27]. Single channel studies revealed that the open probability of RyR2 isolated from aged ventricular myocytes was increased, but with a shorter mean open time, which explains the increase in spark activity. The authors posited that posttranslational modifications of RyR2 may increase the sensitivity to activating Ca^2+^. 

Dynamic and reversible posttranslational modifications of RyR2 are key to modulating channel open probability and grading SR Ca^2+^ release during changing metabolic demands. Phosphorylation of cardiac ion channels and proteins during β-adrenergic stimulation, including RyR2, is well established to increase positive chronotropic and inotropic effects on cardiac function [29,30]. Associated kinases of the RyR2 complex include protein kinase A (PKA), Ca^2+^/calmodulin-dependent kinase II (CaMKII), and associated phosphatases include protein phosphatase 1, 2A, and 2B (PP1, PP2A, and PP2B, respectively). The altered phosphorylation status of RyR2 and changes in the activity of associated kinases and phosphatases have long been implicated in aberrant Ca^2+^ cycling, and this has been well documented in many animal models of cardiac disease, as well as in humans [29,30]. Our current understanding is that both maximum phosphorylation and incomplete dephosphorylation of RyR2 can result in increased activity of the channel [30]. An augmented SR Ca^2+^ leak that is worsened by these modifications can initiate triggered activity and premature ventricular contractions (PVCs), which may degenerate into polymorphic/bidirectional ventricular tachycardia/fibrillation. Since β-adrenergic signaling is deranged in the aging heart [6], one might expect to see reduced PKA-mediated RyR2 phosphorylation. Cooper et al. (2013) reported unchanged RyR2 phosphorylation at PKA-specific sites Serine-2808 and Serine-2031 under basal conditions in aged ventricular myocytes from female rabbits [9], and there are limited data regarding phosphatase association and activity on RyR2 in cardiac aging. The relevance of PKA-mediated phosphorylation of RyR2 to an enhanced SR Ca^2+^ leak remains debated [21,31,32,33]. 

On the other hand, there is substantial evidence to support the role of CaMKII-mediated phosphorylation in modulating RyR2 channel function in both health and disease [34,35,36,37,38]. Cooper et al. (2013) also demonstrated that, in the presence of β-adrenergic agonist isoproterenol, RyR2 phosphorylation at CaMKII site Serine-2814 was significantly increased in ventricular myocytes from old rabbit hearts (four to six years old) in comparison to young (five to nine months old) [9]. A significantly increased activity of stress response kinase JNK2 was reported to activate CaMKII and, thus, upregulate RyR2-mediated diastolic SR Ca^2+^ leak in aged mouse atria (24–32 months old) [39]. Guo et al. (2014) observed increased RyR2 phosphorylation at CaMKII-specific site S2814 in aged mouse atrial myocytes, which increased aberrant intracellular waves and facilitated AF initiation [40]. This was in conjunction with increased oxidation of the channel. 

As another possible posttranslational modification of RyR2, oxidation destabilizes interdomain interactions [41,42,43] and increases open probability of the channel [44,45]. Accelerated SR Ca^2+^ leak via oxidized RyR2s in diseased hearts results in diminished systolic Ca^2+^ release, due to substantial depletion of SR Ca^2+^ content during diastole. At faster pacing rates, this can lead to the occurrence of Ca^2+^-dependent action potential duration alternans. These beat-to-beat fluctuations can contribute as a substrate for arrhythmia [46,47,48,49]. Increased RyR2 activity, in conjunction with accelerated SERCa2a-mediated SR Ca^2+^ uptake during β-adrenergic stimulation, also enhances the propensity for the generation of proarrhythmic, diastolic Ca^2+^ waves [36,45,50,51,52,53,54,55]. Spontaneous Ca^2+^ waves that are large and/or fast enough can evoke NCX1 current that is large enough to reach a threshold for the generation of an extrasystolic action potential, which underlies triggered activity at the organ level [56]. We have previously demonstrated that the age-associated increase of ROS production by mitochondria in aged rabbit ventricular myocytes leads to thiol-oxidation of RyR2, which underlies channel hyperactivity [9]. The formation of advanced glycation end products (AGEs) on proteins is an additional posttranslational modification, first reported to modulate RyR2 in a model of chronic diabetes [57]. Glycation of RyR2 has also recently been documented in myocytes in aged mice (>20 months old) and atrial appendages of elderly patients (>75 years old) [58] and might contribute to enhanced SR Ca^2+^ leak. Increased Ca^2+^ leak due to an altered refractory period for RyR2-mediated SR Ca^2+^ release [51,59,60] contributes to the pathogenesis of triggered activity. Impaired refractoriness and reduced subsequent Ca^2+^ transient amplitude in aged myocytes may also facilitate the onset of cardiac alternans that can drive arrhythmogenesis in the elderly [46,47,48,49]. 

Expression levels of RyR2 in aging are generally reported as unchanged [9,27,61,62,63]. However, as large macromolecular complexes, RyR2 can modulate the altered expression and activity of multiple accessory proteins such as calmodulin (CaM), calsequestrin (CSQ2), triadin, and junctin [64,65]. Most studies to date report no changes in CSQ2 protein expression levels or transcriptional levels [27,62,66], but, more recently, significantly decreased CSQ2 protein levels were observed in aged human atrial myocytes (>75 years old) in conjunction with significantly reduced SERCa2a expression and SR Ca^2+^ content [67]. Reduced CSQ2-mediated buffering of Ca^2+^ within the SR has been reported to cause spontaneous activation of RyR2 [68,69]. The expression and function of other accessory proteins in cardiac aging remain to be explored. 

### 2.2. Sarcoplasmic Reticulum Ca^2+^-ATP-ase

After intracellular Ca^2+^ release, [Ca^2+^]_i_ must be sufficiently reduced for muscle relaxation. Sequestration of Ca^2+^ from the cytosol back into the SR occurs via SERCa2a, which is an ATP-dependent process that restores SR Ca^2+^ content. The affinity of SERCa2a for Ca^2+^ and, thus, its pumping activity is negatively regulated by association with inhibitory protein phospholamban (PLB). Phosphorylation of PLB by PKA or CaMKII relieves this inhibition, which enhances SERCa2a Ca^2+^ affinity and stimulates SR Ca^2+^ uptake [70]. An age-associated reduction in SERCA2a-mediated SR Ca^2+^ uptake is documented in most studies, but is not universal [9,61,71,72]. Reduced pump activity can be due to either reduced expression levels or reduced phosphorylation of the SERCa2a/PLB complex [61,62,66,70,73,74]. Since the SR Ca^2+^ load is a critical factor of cardiac alternans [46], depressed SERCa2a activity in cardiac aging can cause this phenomena, as well as triggered activity, due to increase Ca^2+^ extrusion via NCX1 [75].

Protein expression levels of SERCa2a in aged vs. young myocytes are reported as unchanged [9,53,74] or significantly reduced [66,76,77,78]. A decreased SR Ca^2+^ content and depressed Ca^2+^ transient amplitude was associated with a decrease in SERCa2a and CSQ2 levels in human atrial myocytes (>75 years old) [67]. The expression of PLB is purported to progressively increase with age [79], in which reduces the activity of SERCa2a that may be associated with increased pump inhibition. Schmidt et al. (2005) reported a 40% decrease in SERCa2a protein content in senescent myocardium of rats compared to adult (26 months old vs. 6 months old), with reduced activity [76]. Given that Ca^2+^ efflux into the SR in an ATP-dependent process, SERCa2a overexpression could increase energy requirements in the aged heart. Consumption of ATP by SERCa2a accounts for ~15% of cardiac energy usage [80] and the decreased energy reserve may also contribute to contractile dysfunction in the aged heart. Although SERCa2a overexpression could increase energy requirements of the aged heart, restoration of protein levels by adenoviral gene transfer was shown to normalize diastolic function in vivo, which restores the contractile reserve in aged rat myocardium [76]. Attenuating reduced SERCa2a-mediated Ca^2+^ buffering capacity by gene transfer of parvalbumin also improved diastolic function and force frequency relationship in aged rats [76,81]. 

As β-adrenergic signaling is deficient in the aged heart, this leads to a markedly reduced PKA-dependent phosphorylation of PLB [10,82]. Xu and Narayanan (1998) suggested that an age-related decline in SERCa2a activity may be attributed to altered CaMKII-mediated phosphorylation, given the reduced expression of CaMKII δ isoform in aged rats (26–28 months old) [61]. This would reduce endogenous CaMKII-mediated PLB/SERCa2a phosphorylation, which also reduces pump activity. Altered phosphatase activity may also modulate the phosphorylation status of the SERCa2a/PLB complex, with dephosphorylation of PLB regulated by PP1. Significantly elevated activity of PP1 has been reported in models of heart failure, which is attributable, in part, to reduced levels of its endogenous inhibitor protein I-1 [83,84,85,86,87]. It was shown in the aged mouse heart (20 months old) that inducible expression of constitutively active I-1 increased phosphorylation of PLB at Serine-16/Threonine-17, and this maneuver potentially offset enhanced CaMKII-mediated RyR2 phosphorylation, since incidences of arrhythmia did not increase with age [88]. 

Much like RyR2, SERCa2a is also susceptible to redox modification by free radicals and reactive oxygen species. Knyushko et al. (2005) reported significant accumulation of 3-nitrotyrosine in SERCa2a of aged rat hearts (26 months old) [89]. Declining activity of SERCa2a in aged rat ventricular myocytes could not be explained by differences in protein expression levels of the pump or of PLB, but increased oxidative damage of these proteins was indicated by reduced free sulfhydryl groups [90]. More direct evidence of this modification was shown by Qin et al. [91], whereby hydrogen-peroxide mediated oxidation of SERCa2a at Cysteine-674 contributed to depressed SERCA2a and impaired relaxation of ventricular myocytes in the senescent murine heart (21 months old). Adenoviral overexpression of SERCa2a with this residue mutated to a serine partially preserved SERCa2a activity during H_2_O_2_ exposure.

### 2.3. L-Type Ca^2+^ Channel

Influx of Ca^2+^ into the cytosol serves as the initiating step of intracellular Ca^2+^ release, which activates RyR2 channels by CICR. This occurs at the sarcolemma through LTCCs, which are voltage-dependent and activated during action potential. Regulation of LTCC activity is complex, whereby CaMKII-mediated phosphorylation of C-terminal bound CaM can lessen Ca^2+^-dependent inactivation. Furthermore, channels can be modulated by β-adrenergic stimulation via PKA. Altered activation and inactivation properties of LTCCs can widen the window of current, which increases the propensity of reactivation during late phases of action potential. This can enhance triggered activity in cardiac disease [92]. There are disparate results regarding expression and activity of LTCCs in aging, depending on sex, animal species, and the chamber of the heart [11].

A delayed activation of LTCCs has been suggested to contribute to increased action potential duration in aged rats (24–>27 months old) [93,94,95], as well as greater amplitude of transient outward K^+^ currents (I_to_) [93]. Delayed inactivation and a reduced peak I_Ca_ density was reported in senescent rat ventricular myocytes in another study (>27 months old) [94]. In ventricular myocytes of aged vs. young male mice (24 months old vs. 7 months old), a significant reduction in peak I_Ca_ density as well as significantly slower Ca^2+^-dependent activation was demonstrated, but not in females [96]. Salameh et al. (2010) reported a significant reduction in LTCC maximal conductance and I_Ca_ per cell volume in aged rabbit ventricular myocytes (26 months old) [72]. This effect was compensated by a positive shift in steady state inactivation, which enhanced the late I_Ca_ component. This can induce higher SR Ca^2+^ loading. Decreased I_Ca_ has been observed in atria of aged dogs (>8 years old) [97,98,99,100].

In ventricular myocytes isolated from aged sheep hearts (>8 years old), Dibb et al. (2004) reported a larger peak inward I_Ca_, as well as increased fractional SR Ca^2+^ release and a larger systolic Ca^2+^ transient [101]. No change in excitation-contraction coupling gain (coupling of LTCCs to the release of Ca^2+^ from the SR) was observed in this model, which is suggestive that changes in systolic Ca^2+^ transient arose via increased peak/integrated I_Ca_ rather than due to altered SR content or prolonged action potential duration. This increased I_Ca_ provided increased trigger for RyR2-mediated CICR, while maintaining SR Ca^2+^ content, to sustain cardiac output during increased vessel stiffness and demands of the aged myocardium.

Differences in atrial vs. ventricular LTCC activity with age are evident from a more recent paper by Clarke et al. (2017), whereby atrial myocytes from aged female sheep (>8 years old) showed a decreased peak I_Ca_ [102]. This served to offset an age-associated increase in SR Ca^2+^ content, while enhanced intracellular Ca^2+^ buffering in this model could explain the reduction in Ca^2+^ transient amplitude and the rate of decay of systolic Ca^2+^. Aged sheep are vulnerable for the development of AF, and this is associated with cardiac alternans [103]. 

Reduced expression levels of LTCCs can promote cardiac alternans because of reduced fidelity of coupling with RyR2 channels [104]. Herraiz-Martínez et al. 2015 reported diminished expression levels of LTCC pore forming subunit α1c and, thus, reduced I_Ca_ in aged human atrial myocytes (>75 years old) [67]. However, reduced protein expression levels do not always result in altered I_Ca_. In a study of healthy and failing human hearts, similar I_Ca_ was demonstrated in heart failure despite a significant decrease in expression levels of α1c, which is likely due to enhanced phosphorylation of LTCCs [105]. In cardiac aging, defective β-adrenergic signaling may lead to reduced stimulation of I_Ca_ through PKA [4]. 

The fidelity of LTCC coupling with RyR2 channels and the efficiency of Ca^2+^ release initiation is not only dependent on expression levels, but on the distribution of channels within the myocyte. Primarily located in T-tubules in junctional SR, LTCCs form clusters that oppose clusters of RyR2 channels, and changes in T-tubule structure and function have been implicated in impaired contractility observed in cardiac disease [106,107,108,109]. In ventricular myocytes of aged mice (24 months old), Kong et al. (2018) recently reported reduced I_Ca_ density at the T-tubules, but not at the sarcolemma [110], which further highlights the complexity of LTCC regulation in the aging heart.

### 2.4. Na^+^/Ca^2+^ Exchanger

Although SERCa2a sequesters Ca^2+^ from the cytosol and replenishes SR Ca^2+^ stores, efflux of Ca^2+^ from the cytosol also occurs via NCX1, which is the main route of extrusion from the cardiomyocyte. Working in reversal mode during the early stage of action potential, intracellular Ca^2+^ influx ‘primes’ RyR2 channels and improves the efficiency of CICR [111]. During the late phases of action potential, NCX1 works in a forward mode to extrude Ca^2+^ from the cytosol. Given its electrogenic properties, NCX1 serves as a regulator of action potential during late phases of repolarization and decay of the intracellular Ca^2+^ transient. In the failing heart, upregulation of NCX1 in heart failure contributes to prolongation of action potential duration, reactivation of LTCCs, and the generation of triggered events [56,112,113]. Increased levels of intracellular [Na^+^] in heart failure also enhances reverse mode activity, which may increase [Ca^2+^]_i_ and, thus, RyR2 activity. It is well established that aging also prolongs action potential and diastole [10], which implicates deranged NCX1 activity. However, reports of NCX1 expression levels and activity in aged myocardium vary. 

Reduced expression levels of NCX1 were reported in the hearts of aged male rats and mice (24 months old) [114,115], while others demonstrated unchanged levels of the protein [62,72,116,117]. Mace et al. (2003) demonstrated that, under conditions of controlled [Ca^2+^] and [Na^+^], advancing age in ventricular myocytes of male rats (27–31 months old) was associated with increased forward NCX1 activity, which contributes to prolongation of action potential duration and arrhythmogenesis [116]. In ventricular myocytes, which are isolated from aged sedentary female rats (24 months old), both caffeine-induced Ca^2+^ transients and integrated NCX1 current were increased in comparison to young controls, contributing to diminished contractile function [118]. 

### 2.5. Sexual Dimorphism of Intracellular Ca^2+^ Release

While the risk for cardiovascular disease grows with aging in both sexes, there remains a predisposition to different cardiovascular disease between men and women, with evidence of sex-specific variation in cardiac remodeling [119]. This variation is not only in the morphological structure and vasculature of the aged heart [120], but in Ca^2+^ homeostasis at the cellular level [11]. For an extensive review of sexual differences in cardiac aging and excitation-contraction coupling, see Feridooni et al. [11]. 

Most aging studies described in this review utilized male rodents, whereby the systolic Ca^2+^ transient amplitude was generally reported to decline [27,79,91,96,121]. However, this is not universal [28,122]. Conversely, the Ca^2+^ transient amplitude does not appear to decline with age in female rodents [9,96,121,123], and is demonstrated to increase in aged female sheep [101]. Age-related alterations in excitation-contraction coupling, including peak I_Ca_ density, fractional shortening, and SR Ca^2+^ content were demonstrated to be more prominent with age in male mouse hearts vs. female [96,121]. This sexual dimorphism may aid in explaining why myocardial contractility appears to be better preserved in females in comparison to age-matched males [124]. Age-associated changes in cardiac excitation-contraction coupling proteins are summarized in Table 1. Although differences in intracellular Ca^2+^ homeostasis between aged males and females are apparent, an increase in mitochondrial ROS has been demonstrated in the senescent heart of both sexes [125].

### 2.6. Effects of Mitochondria on Intracellular Ca^2+^ Release

The heart is particularly vulnerable to mitochondrial dysfunction given the huge energetic needs of the contracting myocardium [126]. Cardiac aging is associated with a decline in mitochondrial function, a diminished capacity to maintain redox balance, and an increased emission of ROS [127]. Mitochondria-derived ROS are well established to alter the function of multiple ion channels [29,128] including RyR2 [9,36,41,129], SERCa2a [91,130], LTCCs [131], and NCX1 [132]. 

As evident from the previous discussion, an altered redox status of excitation-contraction coupling proteins appears to be a common mechanism of dysfunction in cardiac aging. It is well established that mitochondrial-mediated oxidative stress contributes to the derangement of cardiac Ca^2+^ homeostasis outlined in this review and it is implicated in the development of both atrial [133,134] and ventricular fibrillation [135]. Scavenging mitochondrial ROS or improving antioxidant activity, therefore, remains an attractive therapeutic strategy in cardiac disease [136]. Mitochondrial-targeted scavenger XJB-5-131 attenuated age-induced loss of cardio-protection and enhanced resistance to IR injury in aged rats (29 months old) [137], while mitoTEMPO reduced oxidative stress in both senescent rat (24 months old) and rabbit (4 to 6 years old) ventricular myocytes [9,138]. Genetic enhancement of mitochondrial antioxidant activity via overexpression of catalase reduced oxidative modifications and attenuated age-related changes in excitation-contraction coupling protein expression [139,140]. Electron and ROS scavenging or inhibition of mitochondrial oxidase has been shown to improve intracellular Ca^2+^ handling and reduce arrhythmic potential in other disease models as well as aging, including heart failure and diabetic cardiomyopathy [9,12,45,141,142]. 

Diminished mitochondrial function and enhanced ROS emission has been attributed to changes in the mitochondrial matrix [Ca^2+^] ([Ca^2+^]_m_). Both increased and decreased [Ca^2+^]_m_ are reported as deleterious to cardiac function [143,144,145,146,147,148,149,150]. Discrepancies in the data may stem from different methods used by experimentalists to measure [Ca^2+^]_m_, including harsh procedures to isolate mitochondria, or loading myocytes with membrane-permeable dyes that can impair membrane integrity. The development of mitochondrial-targeted genetic Ca^2+^ probes and *in vivo* delivery methods should help resolve ongoing controversies [55,143,151,152,153,154]. Utilizing one such probe, we recently demonstrated that enhancement of mitochondrial Ca^2+^ accumulation increased mitochondrial ROS production and enhanced proarrhythmic spontaneous Ca^2+^ release in a rat model of hypertrophy [55]. On the contrary, inhibition of mitochondrial Ca^2+^ influx attenuated pro-arrhythmic activity in this model and reduced mitochondrial ROS emission. In pathophysiology, it would be rational to reduce mitochondrial Ca^2+^ influx, despite some evidence that SR-mitochondria communication may be diminished in aging [58,63,155]. This reduction may be viewed as an adaptive mechanism to reduce mitochondrial [Ca^2+^] and, thereby, limit deleterious mitochondrial ROS production in the senescent myocardium.

## 3. Perspective

Since an explosive growth in the elderly population is expected over the next 20 years [1], it is critical to develop therapies for age-associated cardiovascular disease and to reduce prevalence of sudden cardiac death. It is well established that intracellular Ca^2+^ homeostasis is perturbed in the aged heart, which contributes to increased arrhythmogenesis [10,11]. However, current findings are disparate, depending on species, stage, and sex. These differences must be addressed in future studies and in larger animal models of aging, as well as human tissues. Furthermore, for an improved understanding of the mechanisms that drive Ca^2+^-dependent cardiac dysfunction in the elderly, it is necessary to investigate other proteins that modulate intracellular Ca^2+^ handling including associated accessory proteins, kinases, and phosphatases. 

**Figure 1:**
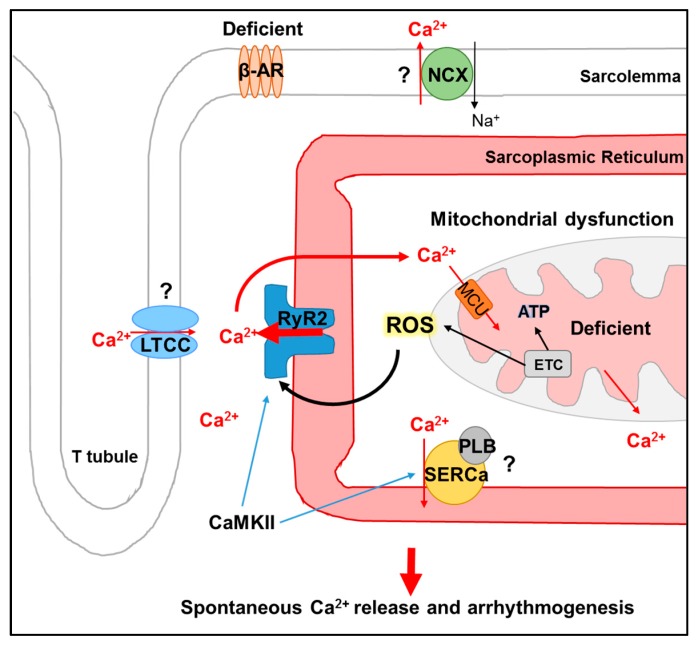
Schematic summarizing the effects of cardiac aging on intracellular Ca^2+^ release in senescent myocytes, via a mitochondrial ROS-RyR2 axis. Virtually universal findings in aged myocytes include (1) deficient signaling through β-adrenergic receptors (β-AR) and (2) mitochondrial dysfunction, including diminished activity of the electron transport chain (ETC) and ATP production, as well an increased ROS emission. (3) It also includes enhanced activity of RyR2, due to oxidation by ROS. CaMKII phosphorylation may also increase RyR2 activity. Increased spontaneous intracellular Ca^2+^ release via oxidized RyR2s underlies arrhythmogenesis. Question marks indicate disparate findings regarding the effects of aging on LTCC, NCX, and SERCa2a/PLB function, whereby activity has been reported as unchanged, increased, or decreased. There is some evidence that mitochondrial Ca^2+^ levels may be diminished in cardiac aging even though this remains to be fully explored.

Although many differences are reported regarding the expression and function of excitation-contraction coupling proteins in the aged heart, virtually universal findings include deficient β-adrenergic signaling, mitochondrial dysfunction, and increased ROS emission, as well as a reduction in intrinsic antioxidant defenses and enhancement of RyR2 activity, regardless of sex [9,26,27,28,40,127,133,134,136]. These universal findings are summarized in Figure 1. Reactive oxygen species have long been identified to play a pathophysiological role in aging, with the theory of free radicals as a primary driving force in determining lifespan introduced in 1956 [156]. It has since been well established that altered redox balance modulates cardiac excitation-contraction coupling [9,36,41,129,130,131,132]. Targeting of mitochondrial ROS and, thus, hyperactive RyR2, therefore, remains an attractive therapeutic target for arrhythmogenesis in cardiac disease and aging [9,55,129,136]. It has been demonstrated that ROS scavenger MitoQ can attenuate ischemia-reperfusion induced cardiac injury [157], hypertrophy [158], and aortic stiffness [159] in animal models of cardiac disease and aging. MitoQ was also shown to improve vascular endothelial function in healthy, older adults [160]. By reducing ROS formation at the mitochondrial respiratory chain, antioxidant peptide SS-31 prevented pressure-overload heart failure in mice [161,162], and many clinical trials with this drug are currently underway (www.clinicaltrials.gov, drug named Elamipretide or MT-131). While these tools hold promise, limited success of ROS scavenging strategies have been reported in most clinical studies [136], which is likely due to insufficient targeting and poor cellular distribution of drugs [136,163]. Given that the balance of ROS production and detoxification is essential to cell function in physiology [164], it also remains unclear as to what level of ROS may be beneficial or detrimental in pathophysiology [136], with some evidence that increased ROS can be beneficial for the function of cardiovascular endothelial cells, depending on source and subcellular localization [165].

An alternative approach to modulating redox balance in aged myocytes is the normalization of mitochondrial Ca^2+^ homeostasis. Oxidative phosphorylation and generation of ATP in the mitochondria is highly dependent on [Ca^2+^]_m_, and there is evidence that mitochondrial Ca^2+^ cycling is altered in cardiac disease [143,144,145,146,147,148,149,150]. Whether reduction or enhancement of [Ca^2+^]_m_ holds therapeutic potential remains controversial [55,143,146,166,167,168], as does the overall contribution of mitochondrial Ca^2+^ to cardiac excitation-contraction coupling [13,169,170,171,172,173]. While we have demonstrated that enhancement of mitochondrial Ca^2+^ accumulation augments RyR2 function and SR Ca^2+^ leak via increased ROS emission [55], there also remains a question as to the reverse: does enhanced SR Ca^2+^ leak via RyR2 directly modulate [Ca^2+^]_m_ and mitochondrial function? Using phosphomimetic mutations of Serine-2808 to modulate RyR2 function, Santulli et al. (2015) suggested that [Ca^2+^]_m_ increases as a result of SR Ca^2+^ leak results in mitochondrial dysfunction in mice [145]. The topology of RyR2 facing the dyad to prime for CICR, rather than the SR-mitochondrial cleft, means only a small amount of Ca^2+^ is likely to be taken up by mitochondria [174], so how much this can be increased under conditions of enhanced leak is unclear. The sensitivity of the mitochondrial Ca^2+^ uniporter (MCU) complex to Ca^2+^ is not thought to be affected by aging [63], and as of yet, there have been few studies investigating mitochondrial Ca^2+^ homeostasis in the senescent heart.

The impact of exercise and physical activity on the progression of cardiac aging is also an active area of research. There is evidence in both healthy animal models and models of other cardiac disease phenotypes that exercise can induce alterations in cardiac Ca^2+^ cycling [175,176,177]. Endurance exercise attenuated increased Ca^2+^ spark frequency, CaMKII-mediated RyR2 phosphorylation at Serine-2814, and Ca^2+^ alternans in a dog model of sudden cardiac death, which reduced ischemically-induced VF [175]. Long-term interval training also reduced CaMKII-dependent phosphorylation of RyR2 and the incidence of VT in a mouse model of CPVT [176]. In spontaneously hypertensive rats, endurance exercise training reversed increased RyR2 expression and attenuated the proarrhythmic increase in Ca^2+^ spark activity in left ventricular myocytes [177]. In the context of the aging heart, research on the effects of exercise on intracellular Ca^2+^ homeostasis remains limited. There is some evidence that exercise training improved Ca^2+^ cycling and contractility, which is associated with increased expression of SERCA2a in aged rodents (28 months old) [178], while others report no change in the expression or function of excitation-contraction coupling proteins in exercise-trained aged rodents [179,180]. In clinical trials, there is evidence that training can improve diastolic function and systolic reserve capacity in the elderly [181,182]. Two years of exercise training in middle-aged adults was recently shown to improve maximal oxygen uptake and decrease arterial stiffness, with authors suggesting this may protect against future cardiac pathologies that are attributable to sedentary aging [183]. There is also modest evidence that exercise improves the blunted response of the aged heart to β-adrenergic stimulation and increases cardiac reserve [184,185]. However, there are several studies that have shown that, while exercise training can improve exercise capacity of the heart, it does not significantly alter cardiac aging phenotypes [185,186,187,188].

Calorific restriction and fasting remains the only strategy demonstrated to significantly increase one’s health span and lifespan, both in animal models and humans [189]. The efficacy of calorific restriction highlights that mitochondrial dysfunction and metabolic derangement may contribute significantly to cardiac contractile dysfunction in the elderly. It has been suggested that sirtuins, which is a subgroup of deacetylases that are expressed in many tissues including the heart, mediate the anti-aging effects of calorific restriction [190]. Protein acetylation is a post-translational regulatory mechanism known to play roles in autophagy, ROS emission, and cell death [191,192]. Reduced expression of sirtuins and increased protein acetylation can result in enzymatic dysfunction and this is associated with multiple chronic and cardiac diseases [193,194,195,196]. Extensive lysine acetylation of mitochondrial proteins has been observed in mouse models of heart failure, as well as in the end-stage human failing heart [197]. Acetylation of excitation-contraction coupling proteins and downstream effects remains relatively unexplored. Application of Sirtuin 1 activator resveratrol enhanced the expression and activity of SERCa2a, which attenuated depressed contractile function in both a mouse model of diabetic cardiomyopathy and a rat model of hypertrophy [198,199]. Acetylation of SERCa2a was recently demonstrated to directly modulate pump function, with elevated acetylation observed in both animal and human failing hearts [200]. There remains limited knowledge of the effects of acetylation and sirtuins on intracellular Ca^2+^ handling proteins and arrhythmogeneses in the aged heart, but this is worthy of future investigation. 

## Figures and Tables

**Table 1:** Changes in excitation-contraction coupling proteins during cardiac aging. Abbreviations: mo—months old, yrs—years old.

Change in Function	Species	Sex	Ages Studied	Myocyte Type	Comments	Reference
**Ryanodine Receptor (RyR2)**						
↑	Mouse	Both	6 vs. 24 mo (young adult vs. old)	Ventricular	Increased spontaneous Ca^2+^ spark activity	[26]
	Mouse	Unreported	6 vs. 24 mo (young adult vs. old)	Ventricular	Increased single channel open probability, increased spontaneous Ca^2+^ spark activity	[27]
	Mouse	Male	3–4 vs. 24–26 mo (young vs. old)	Ventricular	Increased RyR2-mediated diastolic sparks and waves	[28]
	Rabbit	Female	5–9 mo vs. 4–6 yrs (young adult vs. old)	Ventricular	Increased oxidation and SR Ca^2+^ leak, unchanged PKA- but increased CaMKII-mediated phosphorylation	[9]
	Mouse	Male	4–6 vs. >20 mo (young adult vs. old)	Atrial	Increased glycation	[58]
	Mouse	Male	2–2.5 vs. 24–32 mo (young vs. old)	Atrial	Increased JNK2/CaMKII activity, enhanced SR Ca^2+^ leak	[39]
	Mouse	Male	4–5 vs. 24 mo (young adult vs. old)	Atrial	Increased CaMKII-mediated phosphorylation and oxidation	[40]
	Human	Both	<75 vs. >75 yrs (adult vs. old)	Atrial	Increased glycation	[58]
	Human	Both	<55, 55–74, >75 yrs (young, middle aged, old)	Atrial	Reduced CSQ2 expression and increased spontaneous RyR2 activity	[67]
**Sarco-endoplasmic reticulum Ca^2+^-ATPase (SERCa2a)**						
↔	Rabbit	Female	5–9 mo vs. 4–6 yrs (young adult vs. old)	Ventricular	Unchanged protein levels	[9]
	Rat	Unreported	3 vs. 6 mo (young vs. adult)	Ventricular	Unchanged protein levels	[53]
	Rat	Male	5, 15, 26 mo (young adult, adult, old)	Ventricular	Unchanged mRNA levels	[74]
	Rabbit	Male	6 vs. 26 mo (young vs. adult)	Atrial and ventricular	No change in protein expression	[72]
↓	Rat	Male	6 vs. 26 mo (young vs. adult)	Ventricular	Reduced protein expression and pump activity	[76]
	Rat	Male	6–8 vs. 26–28 mo (adult vs. old)	Ventricular	Reduced PKA-dependent PLB phosphorylation	[82]
	Mouse	Male	5, 24, and 34 mo (young adult, old, senescent)	Ventricular	Increased PLB expression	[79]
	Rat	Male	6–8 vs. 26–28 mo (adult vs. old)	Ventricular	Depressed activity associated with reduced CaMKII expression	[61]
	Rat	Male	5 vs. 26 mo (young adult vs. old)	Ventricular	Increased 3-Nitrotyrosine modification	[89]
	Rat	Male	2–26 mo (young-old)	Whole heart	Oxidative damage associated with reduced activity	[90]
	Mouse	Unreported	5 vs. 21 mo (adult vs. old)	Ventricular	Increased SERCa2a oxidation	[91]
	Human	Both	<55, 55–74, >75 yrs (young, middle aged, old)	Atrial	Decreased expression levels associated with reduced SR Ca^2+^ content	[67]
**L-Type Ca^2+^ Channel (LTCC)**						
↔	Mouse	Female	7 vs. 24 mo (adult vs. old)	Ventricular	No alterations in activation or peak I_Ca_	[96]
	Rabbit	Female	5–9 mo vs. 4–6 yrs (young adult vs. old)	Ventricular	No change in peak I_Ca_, reduced responsiveness to β-adrenegic stimulation	[9]
↓	Mouse	Male	3 vs. 24 mo (young vs. old)	Ventricular	Reduced I_Ca_ density at T-tubules	[110]
	Rat	Male	2–3, 8–9, 25–26 mo (young, middle aged, old)	Ventricular	Delayed activation	[93]
	Rat	Male	6 vs. >27 mo (adult vs. old)	Ventricular	Delayed inactivation and reduced peak I_Ca_ density	[94]
	Rat	Male	3, 6–8, 24 mo (young, adult, old)	Ventricular	Delayed activation	[95]
	Mouse	Male	7 vs. 24 mo (adult vs. old)	Ventricular	Slower activation and reduced peak I_Ca_	[96]
	Rabbit	Male	6 vs. 26 mo (young vs. adult)	Ventricular	Reduced I_Ca_ and maximal conductance, enhanced late component	[72]
	Sheep	Female	18 mo vs. >8 yrs (young vs. old)	Ventricular	Increased peak/integrated I_Ca_	[101]
	Dog	Unreported	2–5, >8 yrs (adult vs. old)	Atrial	Reduced I_Ca_, increased I_to_	[97]
	Dog	Both	2–5, >8 yrs (adult vs. old)	Atria	Decreased mRNA and protein expression levels, reduced I_Ca_	[98]
	Dog	Unreported	1–3, >8 yrs (adult vs old)	Atria	Decreased mRNA and protein expression levels, lower peak I_Ca_ density	[99,100]
	Sheep	Female	18 mo vs. >8yrs (young adult vs. old)	Atrial	Decreased peak I_Ca_	[102]
	Human	Both	<55, 55–74, >75 yrs (young, middle aged, old)	Atrial	Decreased peak I_Ca_	[67]
**Na^+^/Ca^+^ exchanger (NCX1)**						
↔	Rats	Male	6 vs. 26 mo (adult vs. old)	Ventricular	Unchanged expression levels	[62]
	Rabbits	Male	6 vs. 26 mo (young vs. adult)	Atrial and ventricular	No change in protein expression	[72]
	Rabbits	Female	5–9 mo vs. 4–6 yrs (young adult vs. old)	Ventricular	No change in protein expression	[9]
	Rat	Male	14–15 vs. 27–31 mo (adult vs. old)	Atrial and ventricular	No change in protein expression	[116]
	Mice	Unreported	3 vs. 26–28 mo (young vs. old)	Ventricular	No change in protein levels	[117]
↑	Rat	Male	14–15 vs. 27–31 mo (middle aged vs. old)	Atrial and ventricular	Increased forward activity	[116]
	Rat	Female	3 vs. 24 mo (young vs. old)	Ventricular	Increased integrated current	[118]
↓	Rat	Male	4 vs. 24 mo (young vs. old)	Ventricular	Reduced protein expression levels	[114,115]

## Abbreviations

AF	Atrial fibrillation
CaM	Calmodulin
CaMKII	Ca^2+^/calmodulin-dependent protein kinase II
CPVT	Catecholaminergic polymorphic ventricular tachycardia
CSQ	Calsequestrin
DAD	Delayed afterdepolarization
EAD	Early afterdepolarization
LTCC	L-type Ca^2+^ channel
NCX1	Na^+^/Ca^2+^ exchanger
PKA	Protein kinase A
PP1	Protein phosphatase 1
PP2A	Protein phosphatase 2A
PP2B	Protein phosphatase 2B
ROS	Reactive oxygen species
RyR2	Ryanodine receptor, type 2
SERCa2a	Sarco/endoplasmic reticulum Ca^2+^-ATPase, type 2a
VF	Ventricular fibrillation
VT	Ventricular tachycardia
